# The BET Inhibitor OTX015 Exhibits In Vitro and In Vivo Antitumor Activity in Pediatric Ependymoma Stem Cell Models

**DOI:** 10.3390/ijms22041877

**Published:** 2021-02-13

**Authors:** Tiziana Servidei, Daniela Meco, Maurizio Martini, Alessandra Battaglia, Alessia Granitto, Alexia Buzzonetti, Gabriele Babini, Luca Massimi, Gianpiero Tamburrini, Giovanni Scambia, Antonio Ruggiero, Riccardo Riccardi

**Affiliations:** 1 UOC Pediatric Oncology, Department of Woman and Child Health and Public Health, Fondazione Policlinico Universitario A. Gemelli IRCCS, 00168 Rome, Italy; daniela.meco@guest.policlinicogemelli.it (D.M.); antonio.ruggiero@unicatt.it (A.R.); riccardo.riccardi@policlinicogemelli.it (R.R.); 2 Department of Pathology, Fondazione Policlinico Universitario A. Gemelli IRCCS, Catholic University of Sacred Heart, 00168 Rome, Italy; maurizio.martini@unicatt.it (M.M.); granittoalessia95@gmail.com (A.G.); 3 Department of Life Sciences and Public Health, Section of Gynecology and Obstetrics, Catholic University of Sacred Heart, 00168 Rome, Italy; alessandra.battaglia@unicatt.it; 4 UOC Oncological Gynecology, Department of Woman and Child Health and Public Health, Fondazione Policlinico Universitario A. Gemelli IRCCS, 00168 Rome, Italy; alexia.buzzonetti@policlinicogemelli.it (A.B.); gabriele.babini@guest.policlinicogemelli.it (G.B.); giovanni.scambia@policlinicogemelli.it (G.S.); 5 UOC Neurochirurgia Infantile, Dipartimento di Scienze Dell’Invecchiamento, Neurologiche, Ortopediche e della Testa-Collo, Fondazione Policlinico Universitario A. Gemelli—IRCCS, Università Cattolica del Sacro Cuore, 00168 Roma, Italy; luca.massimi@policlinicogemelli.it (L.M.); gianpiero.tamburrini@unicatt.it (G.T.)

**Keywords:** pediatric ependymoma, stem cells, epigenetic therapies, BET proteins, BET inhibitors, OTX015, MK-8628, MYCN, MYC, STAT3

## Abstract

Childhood ependymomas are heterogenous chemoresistant neoplasms arising from aberrant stem-like cells. Epigenome deregulation plays a pivotal role in ependymoma pathogenesis, suggesting that epigenetic modifiers hold therapeutic promise against this disease. Bromodomain and extraterminal domain (BET) proteins are epigenome readers of acetylated signals in histones and coactivators for oncogenic and stemness-related transcriptional networks, including MYC/MYCN (Proto-Oncogene, BHLH Transcritpion Factor)-regulated genes. We explored BET inhibition as an anticancer strategy in a panel of pediatric patient-derived ependymoma stem cell models by OTX015-mediated suppression of BET/acetylated histone binding. We found that ependymoma tissues and lines express BET proteins and their targets MYC and MYCN. In vitro, OTX015 reduced cell proliferation by inducing G0/G1-phase accumulation and apoptosis at clinically tolerable doses. Mechanistically, inhibitory p21 and p27 increased in a p53-independent manner, whereas the proliferative driver, phospho-signal transducer and activator of transcription 3 (STAT3), decreased. Upregulation of apoptosis-related proteins and survivin downregulation were correlated with cell line drug sensitivity. Minor alterations of MYC/MYCN expression were reported. In vivo, OTX015 significantly improved survival in 2/3 orthotopic ependymoma models. BET proteins represent promising targets for pharmaceutical intervention with OTX015 against ependymoma. The identification of predictive determinants of sensitivity may help identify ependymoma molecular subsets more likely to benefit from BET inhibitor therapies.

## 1. Introduction

Ependymomas (EPN) are a molecularly and clinically heterogeneous group of neoplasms which occur throughout the nervous system [1]. In children, EPN represent the second most common malignant brain tumors, with a prognosis still dismal in approximately 50% of the patients [2,3]. The extensive molecular characterization and more in-depth knowledge of the pathogenetic mechanisms of the disease have so far not translated into substantial improvement in clinical practice as treatment remains challenging because of high intratumor heterogeneity and intrinsic chemoresistance [4,5]. In the pediatric cohort, four main molecular subgroups have been discovered, two occurring in the posterior fossa (PF_EPN_A and PF_EPN_B) and two in the supratentorial compartment, ST_EPN_RELA proto-oncogene, NF-κB subunit(RELA) and ST_EPN_YAP), distinguished for biological and clinicopathological characteristic [6,7,8]. PF_EPN_A, which represent the majority of childhood EPN, and ST_EPN_RELA are both associated with a particularly unfavorable outcome and demand more effective treatment options. Seminal publications in the field have documented that epigenetic deregulation is involved in the development and maintenance of EPN, suggesting that epigenetic drugs might have a therapeutic potential against these neoplasms [9,10,11,12,13].

Epigenome imbalance plays a major role in cancer development by silencing tumor suppressor genes and activation of driver oncogenes [14]. Childhood brain tumors, including EPN [15], are thought to derive from alterations of the biological processes involved in the embryonic development of the nervous system, which affect a variety of different cell types, including neural stem cells (SC). Pathways relevant to the SC biology are often epigenetically altered carrying out a “malignant SC reprogramming” that confers sustained self-renewal and abnormal differentiation and occurs at the very earliest stages of tumor progression [16,17].

Epigenome is a dynamic condition between open, transcriptionally active, chromatin and repressive chromatin brought about by different mechanisms, including DNA methylation and histone covalent modifications [14,18,19]. Particularly, histone acetylation results from a balance between “writers” and “erasers” of acetylated signals decoded by “reader” proteins, which direct the assembly of nuclear macromolecular complexes at the specific enhancer/promoter regions of key transcription factors involved in oncogenesis. Specifically, acetyllysine marks at the histone tails are recognized by the bromodomain (BD) and extraterminal (BET) protein family, which includes the ubiquitously expressed BRD2, BRD3 and BRD4 [20,21].

The discovery of the multifaceted roles for BET family proteins in controlling gene transcription and chromatin remodeling has fueled research towards BD ligands with drug-like properties able to competitively occupy the acetyl-binding pockets, thus preventing the recruitment of BRD2/BRD3/BRD4 to their cellular targets, such as MYC family members [19,22]. Among the BET inhibitors (BETi) that have moved to clinics, OTX015 (MK-8628) is the only one that has been shown to have a good brain-penetrating capability in preclinical models of glioblastoma and medulloblastoma [23,24] and has been used in Phase 1 studies against glioblastoma (https://clinicaltrials.gov, NCT 02296476). OTX015 exerts robust in vitro and in vivo antitumor activity in preclinical models of a wide range of cancers, including hematopoietic malignancies [25], neuroblastomas [26], breast [27] and prostate cancers [28]. The response to OTX105 differs in tumor types encompassing mechanisms that include suppression of cell proliferation and viability, downregulation of MYC/MYCN proteins and MYC-regulated transcriptional programs, loss of stemness features and chromatin remodeling [25,27,29].

Recently, enhancer-associated genes essential for EPN biology have been profiled, suggesting BETi as a therapeutic strategy to remodel transcription in this tumor [30]. Here, we addressed for the first time the antitumor potential of OTX015-mediated pharmacological inhibition of BET/acetylated histone binding in a panel of EPN SC lines. We found that EPN tumors and patient-derived SC lines express BRD2/3/4 proteins and their targets MYC/MYCN. Inhibition of acetylation-mediated protein–protein interaction by OTX015 reduced the proliferation of EPN SC lines and prolonged survival in two out of three intracranial xenograft models. Together, our in vitro and in vivo data indicate BET proteins as a promising target for pharmaceutical intervention against EPN with OTX015. However, further investigation is warranted to fully explore the underlying molecular mechanisms which mediate the antineoplastic effects of OTX015 to identify patients more likely to benefit from OTX015 and other BETi therapies.

## 2. Results

### 2.1. EPN Patient-Derived SC Lines and Tumors Express OTX015 Targets

We established three SC lines (two of them from two patients with PF-EPN-A and one from a patient with an ST-EPN) that grow in a serum-free medium with the epidermal growth factor/fibroblast growth factor [31,32,33]. We also generated derivative subclones by differential selective pressure exerted by omission of mitogens and/or heterotopic selection in vivo, these model systems reflecting tumor subpopulations which adapt to microenvironment constraints, e.g., to nutrient/growth factor deprivation, such as those which occur in necrotic tumor areas [34,35]. Mitogen-independent (MI) EPN cell lines display increased intracranial tumorigenicity associated with constitutive activation of the epidermal growth factor receptor (EGFR) and the signal transducer and activator of transcription 3 (STAT3). Specifically, both subclonal lines EPP-MI and EPP(#1)-MI express the product of the fusion gene *SEC61G-EGFR*, which resembles the oncogenic EGFRvIII found in glioblastoma from both structural and functional standpoints [32].

**Figure 1:**
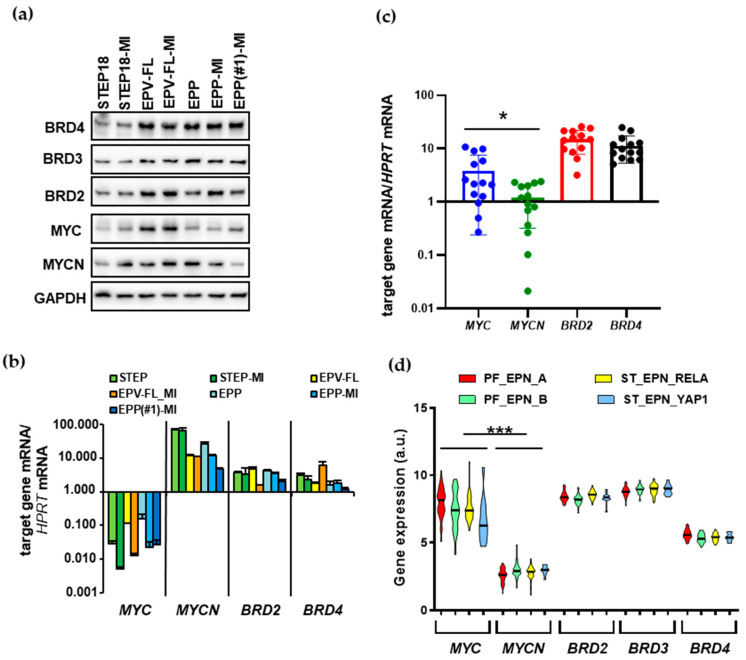
BET proteins and their targets MYC and MYCN are expressed in EPN SC lines and tumors. BRD2/3/4, MYC and MYCN expression at both protein (**a**) and mRNA (**b**) levels were evaluated in EPN cell lines by Western blotting and qPCR, respectively; (**c**) mRNA quantification of *MYC*, *MYCN*, *BRD2* and *BRD4* in 14 pediatric EPN samples; in (**b**,**c**), results are represented as the mRNA levels normalized to the endogenous control hypoxanthine–guanine phosphoribosyltransferase (*HPRT*) in each sample (mean ± SD; *n* = 3); (**d**) mRNA expression of the indicated genes in *n* = 171 intracranial EPN (GSE64415), including PF_EPN_A (*n* = 72), PF_EPN_B (*n* = 39), ST_EPN_RELA (*n* = 49) and ST-EPN-YAP (*n* = 11). Each violin plot represents the distribution of gene expression levels across the samples, and the black line represents the median value. Paired two-tailed Student’s *t*-test was used for statistical significance between *MYC* and *MYCN* expression in (**c**,**d**): * *p* < 0.02; *** *p* < 0.0001. Glyceraldehyde 3-phosphate dehydrogenase (GAPDH).

The expression of BET proteins and of their targets MYC and MYCN was determined at both protein and transcriptional levels in this panel of lines. Western blot analysis showed evident amounts of BRD4, BRD3 and BRD2 proteins in the lines (Figure 1a). Similarly, MYC and MYCN were also expressed across the panel. As for mRNA, qPCR analysis indicated that *BRD2* and *BRD4* mRNA were in a similar range (between one- and six-fold relative to the housekeeping hypoxanthine–guanine phosphoribosyltransferase *HPRT* gene). *MYCN* was expressed at the highest levels of the examined genes, and globally about 400-fold more than *MYC* (Figure 1b).

In order to explore the biological relevance of BET proteins and their targets in EPN, we analyzed mRNA expression of *BRD2*, *BRD4*, *MYC* and *MYCN* in 14 pediatric EPN specimens by qPCR. *BRD2* and *BRD4* mRNA were expressed at a similar extent, whereas global amounts of *MYC* and *MYCN* mRNA were significantly different (*p* < 0.02), though in the pattern opposite to that observed in the lines (Figure 1c). We furthered our analysis in one cohort of EPN tissue samples (GSE64415), focusing on the main four molecular subgroups of childhood intracranial EPN (PF_EPN_A, PF_EPN_B, ST_EPN_RELA, ST_EPN_YAP1). The expression of the selected genes did not show significant biological differences among the EPN subtypes (Figure 1d). In agreement with our qPCR data, the expression of *MYC* was significantly higher compared to that of *MYCN*.

MYCN, but not MYC, is crucial for the phase of precursor cell expansion that precedes the onset of differentiation during the normal nervous system development [36]. Brain tumor SCs, including ependymoma SCs, represent a rare fraction of cells within a tumor that possess the ability to self-renew and generate more differentiated progenies along divergent ganglioneuronal pathways with limited proliferative potential that represent the bulk of the tumor mass [15,37,38,39]. To address whether MYCN/MYC expression might be functionally regulated by differentiation of EPN SC lines, we induced two EPN SC lines to differentiate along the neural lineages as previously described [15,31]. Interestingly, differentiation determined an ~8-fold upregulation of *MYC* mRNA in both lines while strongly downregulating *MYCN* mRNA (Figure S1a). At protein levels, MYCN displayed modest or no induction, while there was a remarkable time-dependent increase in MYC expression accompanied by induction of BET proteins (Figure S1b). Concurrently, non-SC markers of the glial (glial fibrillary acidic protein, *GFAP*), neuronal (tubulin beta 3 class III, *TUBB3*) and oligodendroglial (galactosylceramidase, *GALC*) lineages were significantly upregulated. By contrast, stemness-related genes (oligodendrocyte transcription factor, *OLIG2;* prominin-1, *PROM1*; nestin, *NES*, Sex Determing Region Y-Box 2, *SOX2)* decreased (Figure S1c). It is therefore conceivable that our EPN lines, enriched in stem-like cells, express a high MYCN/MYC ratio, that is switched by differentiation, similarly to what may occur in EPN specimens, where SCs represent only a restricted population of the tumor burden.

Together, these data suggest that BET proteins and their targets MYC/MYCN are expressed in EPN. In addition, because of their modulated expression on the basis of the differentiation state of EPN cells, they may represent potential actionable targets in both the SC and non-SC compartments.

### 2.2. The BETi OTX015 Hampers Proliferation of EPN SC Lines by Targeting Cell Cycle Checkpoint Proteins p21 and p27

**Figure 2:**
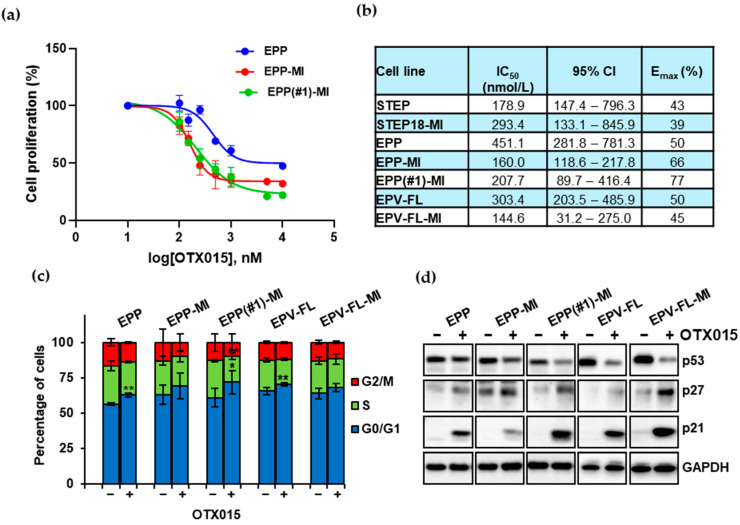
The BETi OTX015 decreases proliferation of EPN cell lines by altering the cell cycle distribution and the expression of regulatory proteins p27 and p21. (**a**) Dose-dependent antiproliferative effects of OTX015 in the indicated lines were determined by cell counting after 72-h exposure. Results represent cell proliferation percentage with respect to vehicle-treated control cells for three independent experiments performed in duplicate (mean ± SD; *n* = 6). (**b**) IC_50_ values with 95% confidence intervals; Emax (%) indicates the maximum inhibitory response induced by OTX015 on cell proliferation. (**c**) Cell cycle progression of the indicated EPN lines was evaluated after 24 h of exposure to 500 nM OTX015. The percentages of the total cell population in the different phases of the cell cycle were assessed by flow cytometry. Each bar represents the mean ± SD from three independent experiments (* *p* < 0.05; ** *p* < 0.01; mean ± SD). (**d**) Western blot analysis of total lysates from EPN cells treated with a vehicle or 500 nM OTX015 for three days. Blots were probed with the indicated antibodies. Glyceraldehyde 3-phosphate dehydrogenase (GAPDH) was used as a loading control.

Next, we evaluated the effects of BET inhibition on proliferation of the EPN SC lines by assessing cell viability after 72 h of exposure to OTX015. The BETi was effective in a dose-dependent manner in all lines, where IC_50_s in a submicromolar range achievable in the clinical setting [40] were observed (Figure 2a, Figure S2a,b). The median IC_50_ was 193 nmol/L (range: 121,7 nmol/L to 451,1 nmol/L) (Figure 2b), similar to the IC_50_ values reported in cell lines from other tumors, including glioblastoma and hematologic malignancies [23,25]. In the PF group, the lines with constitutive activation of EGFR and STAT3 signaling (MI lines) were more sensitive in terms of IC_50_s than the corresponding parental ones. The median value of Emax that indicates the maximum effect corresponding to the minimum measured viability [23] was 50% (range: 39% to 77%). ST lines yielded the lowest Emax (~40%). No correlations were found between OTX015 sensitivity and basal expression of BET proteins, MYC or MYCN.

To characterize the mechanism underlying the antiproliferative effect of BET inhibition in EPN, we evaluated the consequences of applying 500 nM OTX015 on cell cycle regulation in the PF cell lines. A 24-h treatment determined modest changes in the distribution of cells in different phases of the cell cycle: a mild increase in the percentage of cells in the G0/G1 phase was seen in all lines, along with a concurrent decrease in the S-phase (Figure 2c).

From a mechanistic standpoint, G0/G1 arrest is associated with upregulation of the inhibitory p21 and p27. To address whether these proteins are targets of BETi treatment in EPN, we assessed whether OTX015 modulates their expression after three days of treatment (Figure 2d, Figure S2b). The cyclin-dependent kinase inhibitors p21 and p27 exhibited increased expression in all the lines of the panel. This process was very likely p53-independent, since the expression of p53 decreased.

### 2.3. OTX015 Causes Apoptotic Cell Death in EPN SC Lines

**Figure 3:**
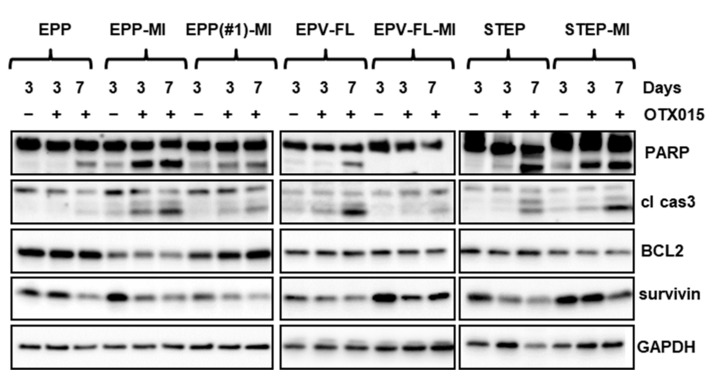
OTX015 triggers apoptosis in EPN cell lines. Western blot analysis of total lysates from EPN cells treated with a vehicle only (−) or with 500 nM OTX015 (+) for three or seven days. Blots were probed with the indicated antibodies. cl cas3 = cleaved caspase 3. Glyceraldehyde 3-phosphate dehydrogenase GAPDH was used as a loading control.

Next, we analyzed the two hallmarks of the apoptotic machinery, i.e., caspase activation and poly (ADP-ribose) polymerase (PARP) cleavage in the panel of lines, after treatment with OTX015 for three and seven days. Both markers increased time-dependently (Figure 3). Relatively little or no changes in the expression of the antiapoptotic BCL2, which is reported as a BET-regulated gene [41], were observed. Interestingly, a consistent finding across the lines was the reduction of the pro-survival oncogenic protein survivin. Overall, the extent and time dependency of the activation of the apoptotic pathway correlated with the sensitivity of cells to OTX015 in terms of Emax, since the most responsive EPP-MI and EPP(#1)-MI lines were the only ones to show early upregulation of cleaved caspase 3 and PARP and concomitant downregulation of survivin. Together, these data indicate that apoptosis contributes to the antitumor activity of OTX015 in EPN.

### 2.4. OTX015 Exerts Minor Effects on the Expression of MYC/MYCN in EPN SC Lines While Markedly Reducing Transcriptionally Active STAT3

We next assessed the time-dependent effects of OTX015 on the expression of its targets. As shown in Figure S3, BRD4 and BRD3 showed minimal changes in the PF lines. In the two ST lines, a marked induction of BRD3 was observed after three days of treatment, whereas by day 7, a decrease in both BRD4 and BRD3 levels was observed in STEP cells. As for BRD2, strong upregulation was reported in STEP and STEP-MI cells by day 3, while a modest increase was documented in EPP and EPP-MI cells, with the protein returning to the basal or even lower levels by day 7 (Figure S3a).

**Figure 4:**
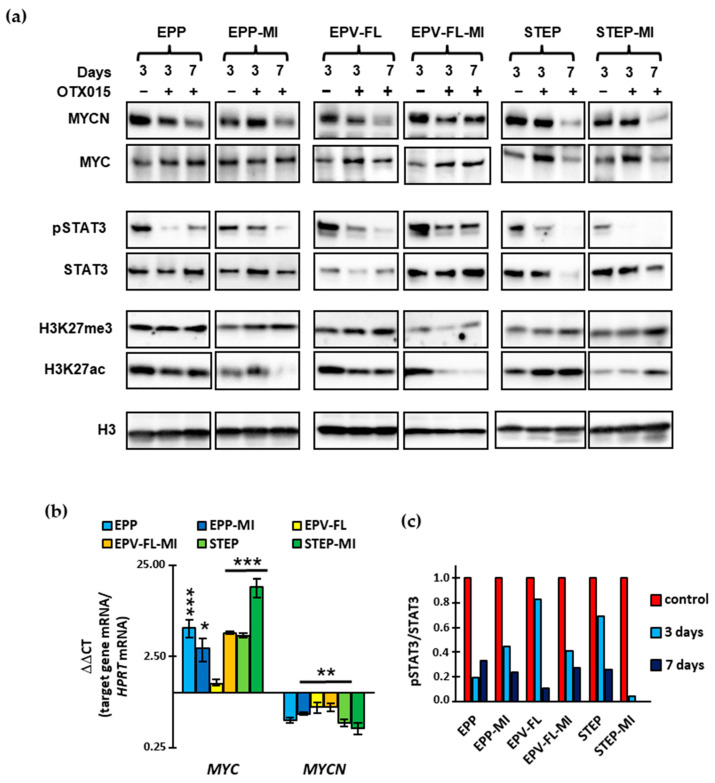
Effects of OTX015 on MYC and MYCN expression and the activation status of STAT3. (**a**) Western blot analysis of total lysates from EPN cells treated with a vehicle (−) or 500 nM OTX015 (+) for three and seven days. Blots were probed with the indicated antibodies. Histone 3 (H3) was used as a loading control. (**b**) qPCR analysis of expression of MYC and MYCN. Levels were normalized to the level of the reference gene hypoxanthine–guanine phosphoribosyltransferase (HPRT) in each sample. Means ± SD (*n* = 3) relative to vehicle-treated controls, which were used as calibrators (1 = no change). Student’s *t*-test was used for statistical significance: * *p* < 0.05; ** *p* < 0.01; *** *p* < 0.001; significantly different from gene expression levels in the vehicle-treated controls. Not significant where no value is indicated. (**c**) Relative changes of phosphorylated STAT3 (pSTAT3) to the corresponding total protein shown in (**a**) were expressed as arbitrary units with respect of the control set to 1 and plotted as bar graphs. Cells treated with a vehicle only for three days were used as the control. OTX015 was used at 500 nM for three and seven days. Densitometry analysis of the bands was performed using the Image Lab software version 6.0.1 (Bio-Rad Laboratories, Inc., Hercules, CA, USA).

OTX015 treatment led to a negative regulation of *MYCN* at the transcript level, while an overt decrease of the protein was observed in all lines only after prolonged exposure to the BETi (Figure 4a,b). By contrast, *MYC* mRNA significantly increased in the majority of lines (5/6), while the protein was slightly upregulated only in EPV-FL-MI, STEP and STEP-MI by day 3, with minor alterations in the other lines and timepoints. Together, these data indicate that both transcriptional and post-transcriptional mechanisms control the expression of MYC and MYCN after exposure to the BETi. Overall, the modulation of the two BET targets did not correlate with cell viability upon exposure to OTX015, indicating that other mechanisms also mediate the antitumor effects of BET inhibition in EPN.

Since it was previously reported that OTX015 affects the STAT3 signaling pathway [25], we analyzed the activation status of this transcription factor in our OTX015-treated cell lines, some of which show constitutive phosphorylation of STAT3. Interestingly, immunoblotting analysis documented a decrease in the transcriptionally active phosphorylated STAT3 (pSTAT3) that was due to inhibition of phosphorylation rather than reduction in the total protein level substantially unchanged (Figure 4a,c).

BET proteins recognize acetylated lysine residues on histone tails and BET inhibition is reported to affect acetylation histone levels [42]. We therefore investigated whether OTX015 could affect the chromatin state by determining active and repressive histone marks, i.e., acetylation of histone 3 lysine 27 (H3K27ac) and three-methylation of histone 3 lysine 27 (H3K27me3), respectively. We found that OTX015 treatment reduced global levels of H3K27ac in PF lines while increasing it in the ST lines. H3K27me3 showed minor or no changes. Together, these data indicate that BET inhibition, in addition to suppressing BET/histone interactions, modulates gene transcription by remodeling the interface between chromatin and BET proteins, as previously reported in glioblastoma models [42].

### 2.5. OTX015 Differentially Alters the Expression of Stemness Genes in EPN SC Lines While Consistently Reducing Vascular Endothelial Growth Gactor A (VEGFA) Expression

In order to investigate whether BET inhibition affects the tumor-initiating ability of EPN SC cultures, we assessed the expression of previously defined neural cancer SC markers (*PROM1*, *NES* and *SOX2*) and of the astrocytic marker *GFAP* in the cells treated with OTX015. As documented by qPCR (Figure S3b), *PROM1* mRNA was significantly reduced only in EPV-FL lines, with no change in the others. A marked decrease in *GFAP* expression was observed in all the lines but EPV-FL-MI and STEP-MI, which exhibited enhanced expression of *GFAP*, *NES* and *SOX2*. Apart from upregulation of *NES* also in EPP and EPP-MI, no other significant modulation of the examined genes was documented. A reduction of the *PROM1* protein CD133 was observed in EPV-FL and EPV-FL-MI lines, as well as in EPP and EPP-MI lines, although to a lesser degree (Figure S3a). No CD133 was detected in the ST lines. Interestingly, mRNA expression of the pro-angiogenetic *VEGFA* was reduced in all cells. Collectively, these results suggest that OTX015 alters the stem-like properties of EPN cells, counteracting the expression of pro-invasive (CD133) and pro-angiogenetic (VEGFA) factors that mediate the interaction of cells with the tumor microenvironment.

### 2.6. Oral Admistration of OXT015 Shows Therapeutic Efficacy against Two out of Three Human EPN Orthotopic Xenografts

We assessed the therapeutic effects of OTX015-mediated BET inhibition in orthotopic EPN models. In the first instance, we compared the in vivo efficacy of the epidrug with that of either the histone deacetylase inhibitor 5-azacytidine (Aza) or carboplatin—a conventional antineoplastic agent used against EPN in clinical practice in orthotopically growing EPP-MI tumors. Groups of animals (*n* = 5) were treated with four therapeutic regimens used in preclinical studies against glioblastoma or EPN xenografts: OTX015 at two dosages [23], Aza or carboplatin [43,44]. Neither carboplatin nor Aza hampered growth of the EPP-MI tumors (Figure S4a). On the contrary, OTX015 significantly prolonged the survival of mice only when used at 50 mg/kg/BID (28 vs. 35 days, respectively, in the control and treated groups, log-rank *p* = 0.02). Aza is currently tested in ongoing clinical studies in EPN (ClinicalTrial.Gov NCT03572530, NCT03206021). Therefore, we compared the antitumor effects of Aza with OTX015 (Figure S4b). However, the combination of the two epidrugs did not substantially prolong the survival of the treated group with respect to the control group. No considerable side effects were observed in the mice treated with any of the drug regimens but carboplatin that caused an almost 25% body weight loss, which was however recovered over time (Figure S4c).

**Figure 5:**
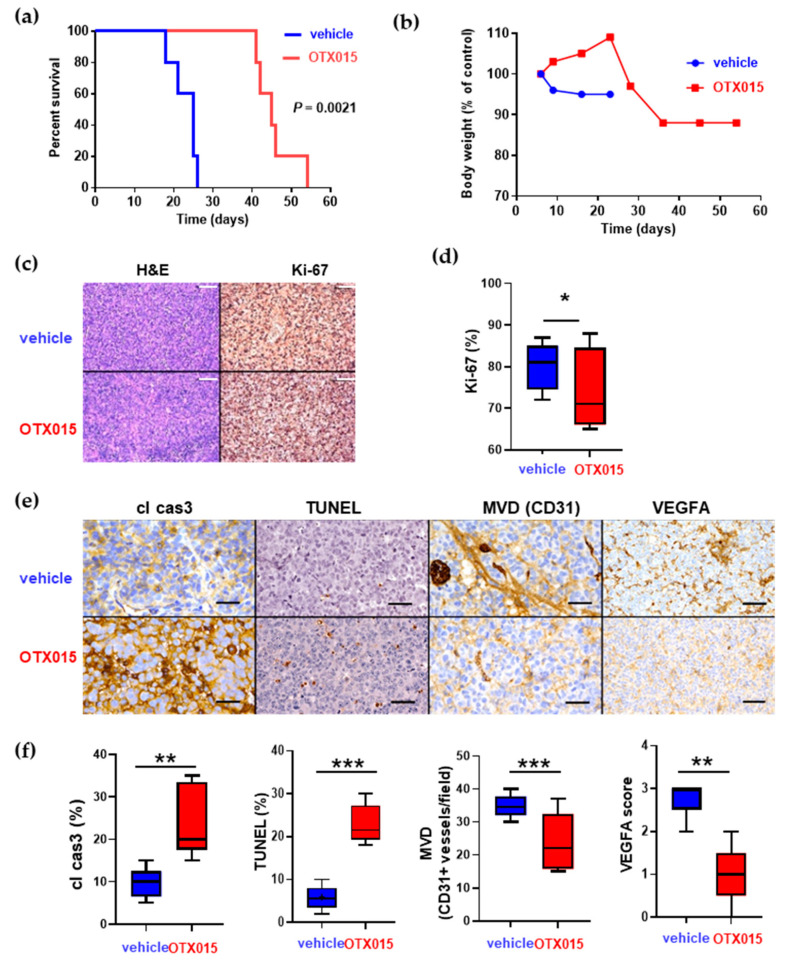
In vivo effects of OTX015 in an EPP(#1)-MI orthotopic EPN model. (**a**) Survival analysis of the mice bearing intracranial EPP(#1)-MI tumors. Animals were treated with a vehicle or OTX015 (50 mg/kg/BID, *n* = 5 mice/group or more). On the appearance of brain tumor symptoms, animals were sacrificed. Survival was examined using the Kaplan–Meier method. (**b**) Effects of the treatment regimen shown in panel (**a**) on the body weight changes of animals bearing EPP(#1)-MI tumors. (**c**) Representative images of EPP(#1)-MI tumor sections stained with hematoxylin/eosin and immunohistochemical staining for proliferating cells (Ki-67), apoptotic cells (cl cas3) and VEGFA. Scale bars, 100 μm. (**d**) Quantitation of staining shown in (**c**) was calculated from three parallel slides (five random fields per each slide) and was expressed as the average percent positive. (**e**) Intracranial EPP(#1)-MI tumor sections from mice treated with a vehicle or OTX015 were stained to detect apoptosis (cl cas3 and TUNEL) and angiogenesis (CD31 and VEGFA). Scale bars, 70 μM. (**f**) Quantitation of staining shown in (**e**) was calculated as the ratio of cl cas3- or TUNEL-positive cells to total cells. The number of CD31-positive vessels/field of tumor sections was used to indicate microvessel density (MVD). The VEGFA score was calculated as described in Materials and Methods. Student’s *t*-test was used for statistical significance. * *p* = 0.05; ** *p* < 0.02; *** *p* < 0.01, significantly different from the sections of vehicle-treated animals. cl cas3 = cleaved caspase 3.

In order to further validate the therapeutic potential of BET inhibition in EPN, we administered OTX015 at the effective schedule to two other groups of mice bearing intracranial EPP(#1)-MI (Figure 5a) or EPV-FL-MI (Figure S5a) tumors. Treatment resulted in an extension of the median survival from 25 to 45 days (log-rank *p* = 0.0021) in the EPP(#1)-MI model, while it was completely ineffective against the EPV-FL-MI xenografts. No signs of toxicity were observed (Figure 5b and Figure S5b).

Because of the higher antitumor activity in EPP(#1)-MI xenografts, we investigated the biologic effects of BET inhibition in formalin-fixed specimens of these tumors when animals were sacrificed on the appearance of brain tumor symptoms. Histopathologic examination of intracranial sections documented that there was a small, though significant reduction in the proliferating fraction (Ki-67 staining) in drug-treated vs. vehicle-treated animals (Figure 5c,d). By contrast, a much more prominent increase in cleaved caspase 3 and terminal deoxynucleotidyl transferase dUTP nick end labeling (TUNEL) staining was documented (Figure 5e,f). Interestingly, treated sections exhibited negative or weak VEGFA staining in comparison to control sections, where moderate or intense membrane staining was observed [45]. Microvessel density (MVD) measured on the basis of CD31-positive endothelial cells in tumor sections is a surrogate marker for tumoral angiogenetic activity. Consistently with our VEGFA data, CD31-immunostained sections from treated animals showed a decrease in newly formed vascular structures (Figure 5e,f). Together, these data suggest that OTX015 exerts antitumoral effects by a proapoptotic/antiangiogenetic rather than an antiproliferative mechanism.

## 3. Discussion

Remodeling transcription by BET inhibition has emerged as an anticancer strategy in a wide range of diseases [46]. Here, we addressed for the first time the therapeutic potential of the brain-penetrating BETi OTX015 in preclinical EPN models. OTX015 treatment at clinically tolerable concentrations compromised cell proliferation in a panel of EPN SC lines which differ in genetic background, EGFR and STAT3 activation and biological behavior in vitro and in vivo [31,32]. Notably, IC_50_ values ranged from 121.7 nmol/L to 451,1 nmol/L, well below the concentrations achieved in patients at tolerable OTX015 doses [23,40]. The IC_50_s in the EPN panel of lines are comparable to concentrations described in other diseases, such as glioblastoma and hematological malignancies [23,25]. In MYC-driven medulloblastoma models, OTX015 IC_50_ between 142.6 nM and 448.6 nM have been reported [24], while in MYCN-driven neuroblastoma, IC_50_s span from 37 nmol/L to >1 μmol/L [26]. Sensitivity to BET inhibition is correlated to amplification and/or overexpression of the respective driver genes in both medulloblastoma and neuroblastoma, i.e., MYC and MYCN, respectively [24,26,47]. However, even in some MYCN-amplified BETi-sensitive neuroblastoma lines, a portion of cells survive the treatment, as documented by the Emax values (range: 55–100%), with some MYCN-amplified lines described as BETi-resistant [48].

The antiproliferative OTX015 effects in EPN were mediated by a decrease in the progression rate from the G1 to the S phase, with a concomitant upregulation of the key cell cycle regulators p21 and p27. In addition to the cytostatic effect, the BETi was able to reduce EPN cell survival by triggering the apoptosis-related pathway and concomitant downregulation of survivin, which correlated with cell line OTX015 sensitivity in terms of Emax.

Cell growth arrest and apoptosis appear to be the main mechanisms by which BET displacement from acetylated signals inhibits various types of cancers, including a range of pediatric tumors [24,26,47,48,49]. Both cytostatic and cytotoxic effects of BET inhibition have been placed in the context of functional p53 [25,41]. However, siRNA-mediated knockdown of p53 does not counteract the induction of p21 and cell loss in response to BETi in glioblastoma cells [41]. Similarly, the ability of OTX015 to compromise viability of triple-negative breast cancer (TNBC) lines is not affected by the presence of mutated p53 [27]. In all our EPN cell lines, p53 was reduced upon exposure to OTX015, suggesting that the antiproliferative/proapoptotic response to BET inhibition does not uniquely rely on p53 in EPN, with potential clinical implication of BETi even in the subset of tumors with inactivated p53, a frequent finding in ST_EPN_RELA [50].

From a mechanistic standpoint, the anticancer effects of BETi have been linked to their transcriptional repression of so far “undruggable” key oncogenes, including MYC/MYCN and their downstream target genes [26,48,49,51]. MYC/MYCN oncoproteins are master regulatory factors for fundamental cellular processes [52]. Hence, MYC- and MYCN-driven tumors have been regarded as targets for anticancer therapy with BETi [53]. Although there is some debate over the identification of biomarkers of susceptibility towards BET inhibition, MYCN and MYC expression remains the most common genetic predictor of sensitivity to BETi.

In EPN, we found that OTX015 determined a decrease in *MYCN* mRNA, whereas the protein was reduced only after prolonged exposure. MYC showed no changes or mild augmentation at most in spite of a marked increase in mRNA. Together, these data highlight both transcriptional and post-transcriptional regulation of MYC/MYCN by BETi in EPN, as previously reported in acute myeloid leukemia [54]. In addition, because of the overall modest modulation of these transcription factors, very likely they are not the main BET targets in EPN.

In support of our finding, mechanisms other than MYC/MYCN downregulation contribute to BETi activity, because inhibition of cell proliferation has been reported even with no reduction, or even an increase, of these transcription factors in lines from different tumor types [23,27,41]. In preclinical TNBC models, the antitumor effects of OTX015 appear to be mostly MYC-independent. Indeed, significant and durable post-OTX015 downregulation of the MYC protein and mRNA was observed only in one out of the three lines examined at equivalent growth-inhibiting effects [27]. Consistently, a TNBC OTX015-responsive xenograft model showed no alteration in the expression of MYC with respect to the vehicle-treated group. Similar data have been documented in glioblastoma [23,41] and lung adenocarcinoma models [55], where antiproliferative effects and transcriptional changes by BET inhibition occur at unchanged MYC/MYCN levels. In agreement with MYC-independent mechanisms of BET inhibition that take place in some tumor contexts, ectopic overexpression of MYC/MYCN is not able to abrogate the suppressive activity of BETi in neuroblastoma [26] and glioblastoma lines [41].

In SC biology, MYC family members play a pleiotropic role, encompassing the maintenance of self-renewal and pluripotency, cell identity and the commitment to terminal differentiation [52,53,56,57]. MYCN, but not MYC, is required for the expansion of progenitor cells in the developing central nervous system. We found that EPN SCs express a high ratio of *MYCN*/*MYC* mRNA that is switched by differentiation concurrently with the upregulation of glioneuronal markers and the downregulation of stemness-related markers. It is tempting to speculate that *MYCN* expression is restricted to EPN SCs, although more investigation is needed to fully address the role of MYCN in EPN biology. In agreement with our hypothesis, we found a low *MYCN*/*MYC* mRNA ratio in EPN specimens, where SCs represent a restricted population of the tumor burden [15,37,38]. The interrogation of a specific EPN gene expression dataset confirmed differential expression of *MYC* and *MYCN* irrespective of the molecular subgrouping.

Biological activity of BETi includes, but is not limited to, MYC-mediated transcriptional programs, because effects on other key transcriptional pathways such as E2F and STAT3 have also been documented [25,29]. Here, we provide evidence that OTX015 decreases transcriptionally active STAT3 in all EPN lines, likely through the modulation of upstream cytokines and/or receptor tyrosine kinases. Our findings are of clinical relevance because of the crucial role of STAT3 in the control of core cancer pathways, including tumor cell proliferation, survival and tumor-promoting inflammation [58]. Several lines of evidence indicate that STAT3 mediates fundamental cellular processes in EPN. Expression of STAT3 is markedly elevated in EPN tissues compared with a control brain [59]. More importantly, a significant increase in activated STAT3 has been reported in the anaplastic histology group (WHO III) with respect to WHO II ependymoma [59]. In line with these findings, a more recent publication showed that persistent STAT3 activation drives growth of high-risk PF_EPN_A [60]. Pharmacological inhibition of activated STAT3 or knockdown with specific STAT3-siRNAs hampers proliferation and increases apoptosis of EPN cells with concomitant downregulation of survivin expression [59,60]. Genes of the STAT3 pathway and of the STAT3 activator IL6 are significantly enriched in group PF_EPN_A, suggesting the establishment of a feedforward loop between tumor cells and the inflammatory microenvironment, which can provide additional druggable vulnerabilities by BETi in EPN. Of note, *VEGFA* and *BIRC5* (which encodes survivin) are both regulated by STAT3 [58], raising the hypothesis that the downregulation that we observed in our panel of EPN cells might be mediated by STAT3 inactivation.

In addition to impacting multiple transcriptional programs simultaneously, treatment with BETi also affects the chromatin state in EPN cells, as evidenced by the modulation of the active histone mark H3K27ac, which is exclusively present at active enhancers [61]. The global reduction in acetylation at histone H3K27 indicates an overall loss of permissive chromatin state in PF lines and thereby of transcription, whereas in ST lines, BET inhibition appears to favor an open chromatin structure. It is therefore possible that BET inhibition globally redistributes BET proteins to chromatin, thus altering the cellular epigenetic landscape and the oncogenic program of EPN cells. Hence, BET proteins might have dual functions in shaping both the epigenome and the transcriptome in EPN. Global H3K27ac depletion after BET inhibition has been reported to occur in embryonic SC, leukemia and glioblastoma cells, specifically at enhancer regions [42,61], implying that the epigenetic marker formation at the regulatory regions is a common mechanism by which BETi exert their biological effects.

Our in vivo data show encouraging results, because oral OTX015 administration significantly extended survival of animals in two out of three orthotopic models. In a comparative study with Aza and carboplatin in the EPP-MI model, OTX015 was the only effective drug. Even superior antitumor activity was observed in the EPP(#1)-MI xenografts, where a survival advantage of 20 days was reached in drug-treated animals. These two models both harbor the *SEC61G-EGFR* fusion [32], which may be one, but not the only molecular determinant of sensitivity to BET inhibition. Indeed, EPP(#1)-MI xenografts, which express the smaller amount of the fusion product (Figure S6), were more responsive to the treatment. Interestingly, EGFRvIII-expressing glioblastoma cells display increased apoptotic responsiveness to the BETi JQ1 and two xenograft lines carrying EGFRvIII mutation are highly sensitive to JQ1 [41,62]. Collectively, our data and data from the literature suggest that EGFR mutants driving oncogenic activation of the receptor-mediated pathway may increase the response to BETi.

BETi have shown promise in multiple therapeutic contexts. Here, we provide evidence that BET proteins play a key role in regulating growth and survival in genetically diverse EPN SC models and OTX015-mediated BET inhibition exerts antitumor effects both in vitro and in vivo. Very recently, BRD3 has been identified as a therapeutic actionable marker to target neuronal lineage precursors in PF EPN [63], whereas MYCN amplification has been found to drive an aggressive form of spinal EPN [64,65], possibly identifying these molecular EPN subgroups as candidates for BETi therapy. However, the identification of other reliable predictive markers of sensitivity to BETi is warranted to improve target-driven therapy and guide effective combination approaches with BETi and other chemotherapeutics in EPN.

## 4. Materials and Methods

### 4.1. Cell Cultures and Reagents

The PF cell lines EPP, EPP-MI, EPV, EPV-FL and EPV-FL-MI were established in our laboratory and previously characterized for stemness-defining features, e.g., expression of neural stemness markers, neurosphere-forming ability, self-renewal, long-term propagation and tumor-initiating capability in nude mice [31,32,33]. All the EPN lines used in this study were grown in 3D conditions in a Neurocult medium ( STEMCELL Technologies, Cambridge, UK) supplemented with 20 ng/mL EGF (Sigma-Aldrich, St. Louis, MO, USA) and 10 ng/mL FGF2 (Promega, Madison, WI, USA), a well-validated culture condition to favor neural stem-like cell growth [15,66]. Derivative mitogen-independent subclones were selected and maintained in the absence of exogenous growth factors as previously described [32]. Cells were maintained in a humidified atmosphere with 5% CO_2_, 5% O_2_. The EPP-MI cells expressed the product of the fusion gene *SEC61G-EGFR*. The EPP(#1)-MI line was generated by MI selection of the very first passage of the patient-derived EPP line in the attempt to establish a line without fusion. However, although low EGFR fusion expression and high wild-type EGFR expression were detected in the very first passages of selection, eventually the pattern switched, further confirming that *SEC61G-EGFR* fusion is an inherent genetic feature of this tumor line (Figure S6a). The newly established ST cell line STEP18 and its MI derivative STEP18-MI did not express *RELA* fusion products according to the Western blot analysis (Figure S6a) [8]. Both lines exhibited neural stemness markers (Figure S6b), neurosphere-forming ability, self-renewal, long-term propagation and tumor-initiating property (Figure S6c) that was increased in the STEP18-MI line. The ST lines showed very low *PROM1* mRNA (Figure S6b), no CD133 protein (Figure S3a) and delayed tumorigenicity in agreement with a previously characterized ST-EPN-RELA line [67]. Differentiation assays were performed as previously described [15]. Briefly, dissociated neurospheres were allowed to attach onto poly-L-ornithine/laminin (Invitrogen Corporation, Carlsbad, CA, USA)-coated vessels. After 24 h, the medium was changed to a Neurobasal medium (Invitrogen Corporation, Carlsbad, CA, USA) with 10% FBS for up to seven days.

OTX015 (from MedChemExpress LLC, Monmouth Junction, NJ, USA), was dissolved in DMSO to a stock concentration of 10 mM for in vitro experiments. Serial dilutions were made in cell culture media prior to cell treatments. For in vivo assays, OTX015 was dissolved in water immediately before administration. Aza (Sigma-Aldrich, St. Louis, MO, USA) was dissolved in water, whereas carboplatin was reserved clinical use.

### 4.2. Patients and Tissue Samples

Surgical EPN specimens and clinicopathologic information were used in accordance with the institutional review board approval (Fondazione Policlinico Gemelli, ID1648; Prot. 44510/17). Eleven tumors were PF_EPN and three were ST_EPN. According to the histopathologic classification, seven tumors were WHO grade II, while nine tumors were anaplastic WHO grade III EPN. The ST cell line STEP18 was generated from a recurrent WHO grade III ST_EPN from a 13-year-old boy in accordance with the institutional review board approval (ID1648; Prot. 44510/17). No molecular characterization for subgroup affiliation of the ST_EPN sample was available.

### 4.3. Cell Viability, Cell Death, and Cycle Analyses

Twenty-four hours after seeding onto 6-well plates, the cells were treated with vehicle DMSO or serial dilutions of OTX015 (range: 0.1 to 10.0 μmol/L) for 72 h. Cell number and viability were assessed by automated cell count (NucleoCounter 100TM, ChemoMetec, Steen Søndergaard, Denmark) as previously described [31]. The drug concentration at which cell proliferation is reduced by half (IC_50_), the 95% confidence interval (95% CI) and maximal effect (Emax, defined as the cell proliferation inhibition percentage at the highest dose) were calculated using the sigmoidal dose–response function (variable slope) of data with the GraphPad Prism version 8.00 for Windows (GraphPad Software Inc., San Diego, CA, USA) as previously published [23]. Analysis of cell cycle perturbation was assessed using the standard flow cytometry method. Briefly, at the end of each incubation period, free-floating clusters of neurospheres were collected and gently disrupted to obtain viable single cell suspensions. Single EPN cell suspensions were then fixed in 1 mL 70% *v*/*v* cold ethanol at the concentration of 1 × 10^6^ cells/mL, adding the ethanol dropwise while vortexing, and incubated at −20 °C for no longer than 10 days. Prior to DNA staining, fixed EPN cells were spun down and treated with RNase (100 µg/mL) for 10 min. The cells were then stained with propidium iodide (PI, 0.05 mg/mL) and maintained at +4 °C overnight. The day after, stained EPN cells were subjected to flow cytometry for cell cycle analysis by quantitation of cellular DNA content using a Beckman Coulter Navios flow cytometer. A minimum of 30,000 cells of interest were acquired for each sample at a low flow rate (<200 events/s). Analysis of cell distribution in different phases of the cell cycle was performed using the MultiCycle AV DNA analysis available in the FCS Express software version 7 (De Novo Software, Los Angeles, CA). Pulse shape processing was used to exclude cell doublets from analysis. A time vs- PI plot was also used to monitor fluidic stability and ensure consistent sample analysis.

### 4.4. cDNA Synthesis and Quantitative PCR (qPCR)

Total RNA was extracted from cell lines or frozen tissues using an AllPrep^®^ DNA/RNA/Protein Kit (Qiagen) according to the manufacturer’s instructions. RNA (1 μg) was retro-transcribed with Superscript III reverse transcriptase and random primers (Thermo Fisher Scientific, Waltham, MA, USA). Gene expression was quantified by quantitative real-time PCR (qPCR) performed on a 7500 Real-Time PCR System (Applied Biosystems, Foster City, CA, USA) using TaqMan gene expression assays (Applied Biosystems). Expression levels of *BRD4, BRD2, MYC*, *MYCN*, *PROM1*, *NES*, *GFAP*, *SOX2*, *VEGFA, OTX2* and *OLIG2* transcripts relative to *HPRT* or *GAPDH* were determined using the threshold cycle method or the ΔΔCt.

### 4.5. Western Blot Analysis

The cells were seeded in 100 mm dishes and challenged 24 h later with 500 nM OTX015 for different time intervals. Vehicle-treated cells were used as controls. At the end of the treatment period, the cells were washed with ice-cold PBS and disrupted in lysis buffer as previously described [31]. Cell lysates (20 µg) were resolved by precast SDS-PAGE gel (Bio-Rad Laboratories, Inc., Hercules, CA, USA) and probed with the antibodies listed in Table S1. All blots were incubated with the appropriate secondary horseradish peroxidase conjugated antibodies and developed by enhanced chemiluminescence (ECL prime, GE Healthcare, Amersham, United Kingdom). Densitometry analysis of the bands was performed using the Image Lab software version 6.0.1 (Bio-Rad Laboratories Inc., Hercules, CA, USA). Uncropped Western blots are reported in Figure S7.

### 4.6. In Vivo Models and Animal Treatment

All experimental animal investigations complied with the guidelines of the Ethical Committee of the Catholic University and of the “Istituto Superiore di Sanita’’ (National Institute of Health, Rome, Italy; OPBA 35-01, Approval No. 243/2015). For intracranial transplantations, 3 × 10^5^ cells/10 μL PBS were implanted into the lateral ventricle of 5-week-old male nude CD1 nu/nu mice (Charles River) as previously described [68]. Six days from the operation (Day 7), mice were randomly divided into groups of five and treated with one of the following: OTX015 (25 or 50 mg/kg/bidaily (BID) po) [23], Aza (5.0 mg/kg intravenously (i.v.), daily for five consecutive days), carboplatin (90 mg/kg once daily, continuously, i.v.) [43,44]. The control group received only a vehicle. Animals were monitored daily until symptomatic, when they were euthanized and brains were removed for histopathologic analysis. Statistical analyses were performed using the GraphPad Prism version 6.0 for Windows (GraphPad Software Inc., San Diego, CA, USA). Survival of animals was determined using the Kaplan–Meier plots and compared by the log-rank test. *P*-values < 0.05 were considered to be significant.

### 4.7. Immunohistochemical Analyses

Immunohistochemical analysis was performed as previously described [45]. Unstained tissue sections (4 μm-thick) were cut from formalin-fixed, paraffin-embedded blocks and mounted on positively charged glass slides. For antigen retrieval, deparaffinized and rehydrated sections were treated with a citric acid buffer (pH 6.0) followed by inhibition of endogenous peroxidase with 3% H_2_O_2_ for 5 min. Then, the sections were incubated overnight at 4 °C with primary antibodies, followed by visualization with the avidin–biotin–peroxidase complex method (UltraTek HRP Anti-polyvalent; ScyTek, Logan, UT, USA). 3,3′-Diaminobenzidine was used as the enzyme substrate to observe the specific antibody localization, and Mayer hematoxylin was used as a nuclear counterstain. Regions of normal brain, which included both the cortex and white matter, were used as internal controls. For quantification of apoptotic cells, an antibody that specifically recognizes the cleaved caspase 3 assay was used. In addition, TUNEL assays were performed with an in situ cell death detection kit, POD (Roche Group, Basilea, Switzerland) according to the manufacturer’s protocol. Staining was quantified as the ratio of positive cells to total cells in each field using five random fields. The staining intensity of tissue slides was evaluated independently by two observers. To assess differences in VEGFA staining intensity, an immunoreactivity scoring system was applied [45]. Intensity of staining was classified by both the percentages of the cells stained and the intensity of staining. In this way, the final scores of 0 to 3 were obtained (0, negative; 1, weak; 2, moderate; 3, strong). Only cells with moderate to strong staining were interpreted as positive. Microvessel density (MVD) was detected using anti-human CD31 mouse antibody as previously described [45]. Briefly, individual microvessel counts were made in areas of most intense neovascularization. Any CD31-positive endothelial cell or endothelial cell cluster was considered as a single countable microvessel. MVD was expressed as the mean number of microvessels per field from three highly vascularized areas in each section.

### 4.8. External Gene Expression Validation

To validate the expression of BET proteins and their targets in an external patient cohort, gene expression data were retrieved from the Gene Expression Omnibus (GEO) database. In particular, GSE64415 dataset included 209 ependymal tumor samples classified by molecular subgroups, and were hybridized to the Affymetrix HG U133 Plus 2.0 microarrays [1]. Hybridization probes for MYC, MYCN, BRD2, BRD3 and BRD4 were identified and the mean intensity values were analyzed in GraphPad Prism version 8.0 (GraphPad Software Inc., San Diego, CA, USA) to compare differences between genes and within the molecular subgroups.

### 4.9. Statistical Methods

Statistical significance for in vitro assays was assessed using the Student’s *t*-test. For in vivo assays, statistical analyses were performed using the GraphPad Prism version 6.0 for Windows (GraphPad Software Inc., San Diego, CA, USA). Survival of animals was determined using the Kaplan–Meier plots and compared by the log-rank test. *p*-values < 0.05 were considered to be significant.

## 5. Conclusions

BET proteins control fundamental cellular processes in multiple tumors, indicating BET proteins as actionable targets for anticancer strategies. In patient-derived EPN stem cell models, OTX015-mediated pharmacological inhibition of BET proteins diminishes cell proliferation in vitro and significantly prolongs survival of mice bearing intracranial tumors in two out of three models. Our encouraging preclinical results pave the way for therapeutic targeting of BET epigenetic readers in EPN. The identification of predictive markers of sensitivity to BET inhibition may help to further develop more effective therapeutic interventions with BETi and select EPN molecular subsets more likely to benefit from epigenetic therapy.

## Data Availability

The data that support the findings of this study are available from the corresponding author upon reasonable request.

## Supplementary Materials

Supplementary Materials can be found at https://www.mdpi.com/1422-0067/22/4/1877/s1.
