# Supplementary material for: The BET Inhibitor OTX015 Exhibits In Vitro and In Vivo Antitumor Activity in Pediatric Ependymoma Stem Cell Models
Source: Int J Mol Sci. 2021 Feb 13;22(4):1877. doi: 10.3390/ijms22041877 (PMC7918371; doi:10.3390/ijms22041877)
Supplement: Supplementary file 1 [file ijms-22-01877-s001.pdf]

International Journal of *Molecular Sciences*

MDPI

*Article*

# The BET inhibitor OTX015 exhibits in vitro and in vivo antitumor activity in ependymoma stem cell models

**Tiziana Servidei [1,*], Daniela Meco [1], Maurizio Martini [2], Alessandra Battaglia [3], Alessia Granitto [2], Alexia Buzzonetti [4], Gabriele Babini [4], Luca Massimi [5], Gianpiero Tamburrini [5], Giovanni Scambia [4], Antonio Ruggiero [1], Riccardo Riccardi [1].**

[1] UOC Pediatric Oncology, Department of Woman and Child Health and Public Health, Fondazione Policlinico Universitario A. Gemelli IRCCS; 00168, Rome, Italy; tiziana.servidei@guest.policlinicogemelli.it (T.S.); daniela.meco@guest.policlinicogemelli.it (D.M.); antonio.ruggiero@unicatt.it (A.R.); riccardo.riccardi@policlinicogemelli.it (R.R.)

[2] Department of Pathology, Fondazione Policlinico Universitario A. Gemelli IRCCS; Catholic University of Sacred Heart; 00168, Rome, Italy; maurizio.martini@unicatt.it (M.M.); granittoalessia95@gmail.com (A.G.)

[3] Department of Life Sciences and Public Health, Section of Gynecology and Obstetrics; Catholic University of Sacred Heart; 00168, Rome, Italy; alessandra.battaglia@unicatt.it (A.B.)

[4] Department of Woman and Child Health and Public Health, Fondazione Policlinico Universitario A. Gemelli IRCCS; 00168, Rome, Italy; alexia.buzzonetti@policlinicogemelli.it (A.Bu.), gabriele.babini@guest.policlinicogemelli.it (G.B.), giovanni.scambia@policlinicogemelli.it (G.S.)

[5] UOC Neurochirurgia Infantile, Dipartimento di scienze dell’invecchiamento, neurologiche, ortopediche e della testa-collo, Fondazione Policlinico Universitario A. Gemelli—IRCCS, Università Cattolica del Sacro Cuore, 00168 Roma, Italy; luca.massimi@policlinicogemelli.it (L.M.); gianpiero.tamburrini@unicatt.it (G.T.)

* Correspondence: tiziana.servidei@guest.policlinicogemelli.it Tel.: +39-06-30155165

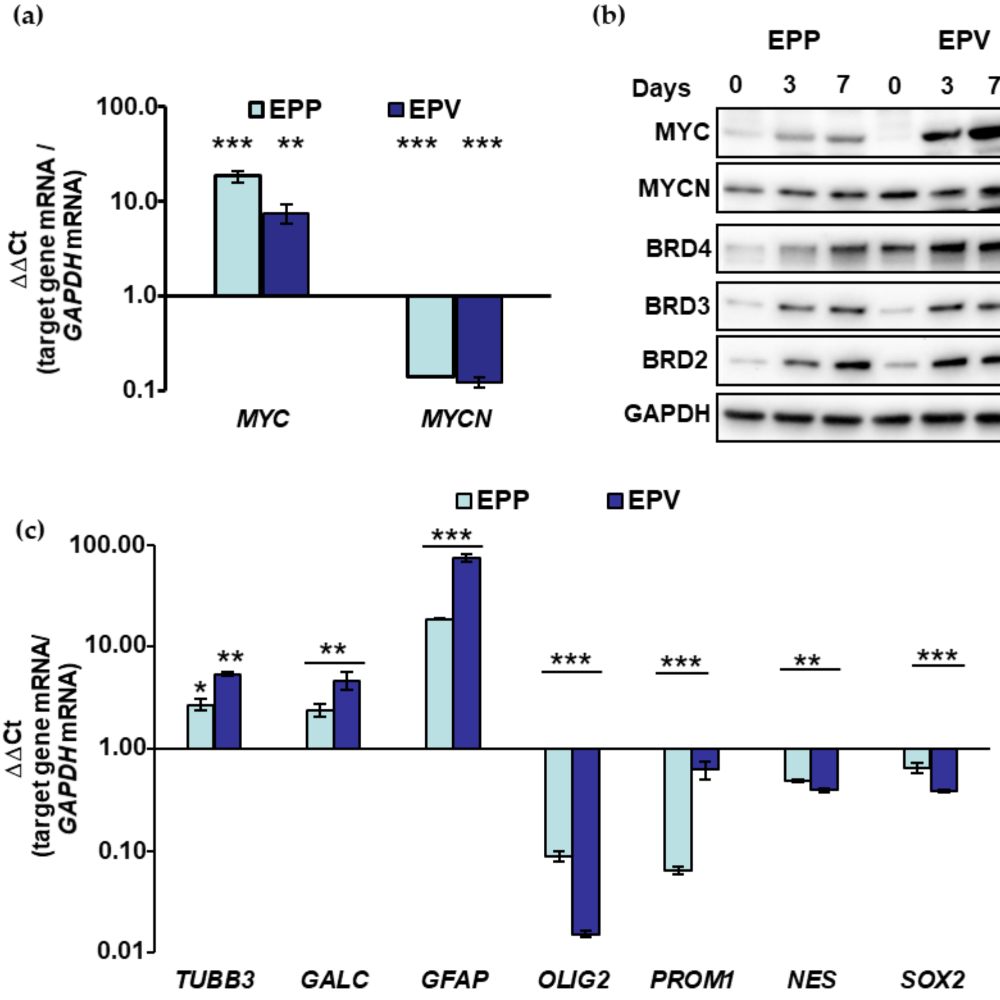


**Figure S1.** MYC and MYCN expression is modulated by differentiation in EPN SC lines. **(a)** EPP and EPV cells were grown in differentiation conditions (10% serum-containing medium without growth factors) for seven days. qPCR analysis of *MYC* and *MYCN* expression was performed and levels were normalized to the level of glyceraldehide 3-phosphate dehydrogenase *(GAPDH)* in each sample. Fold changes were calculated relative to undifferentiated controls by the ΔΔCT method (mean ± SD; n = 3). **(b)** EPP and EPV cells were allowed to differentiate for three and seven days. Cell lysates were subjected to immunoblot analysis with antibodies to the indicated proteins. GAPDH was used as a loading control. **(c)** Fold changes of the expression of non-SC (tubulin beta 3 class III *TUBB3,* galactosylceramidase *GALC,* glial fibrillary acidic protein *GFAP*) and stemness-related (oligodendrocyte transcription factor *OLIG2,* prominin 1, *PROM1,* nestin, *NES,* sex detrming region Y-Box transcription factor 2, *SOX2*) genes were assessed in EPP and EPV differentiated cells by the ΔΔCT method (mean ± SD; n = 3) as described in panel (a). In (a) and (c) unpaired two-tailed Student t test was used for statistical significance: * P < 0.05; ** P < 0.01; *** P < 0.001; significantly different from gene expression levels in the corresponding undifferentiated cells.

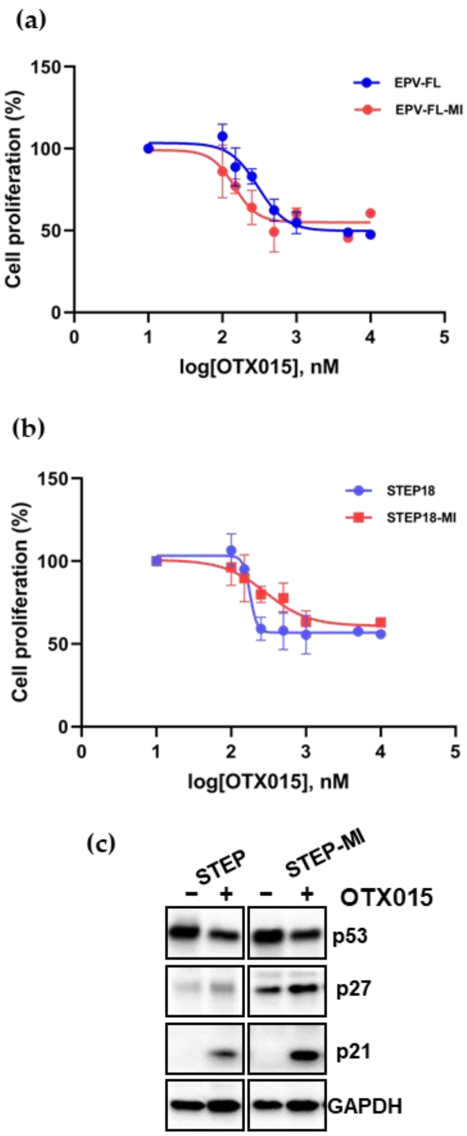


**Figure S2.** BET inhibition decreases proliferation of EPN cell lines by altering the expression of regulatory proteins p27 and p21Cip. Dose-dependent antiproliferative effects of OTX015 in EPV-FL and EPV-FL-MI **(a)**, STEP and STEP-MI **(b)** were determined by cell counting after 72 h exposure. Results represent percent cell proliferation with respect to vehicle-treated control cells for three independent experiments performed in duplicate (mean ± SD; n = 6). **(c)** Western blot analysis of total lysates from STEP and STEP-MI cells treated with vehicle or OTX015 500 nM for three days. Blots were probed with the indicated antibodies. Glyceraldehide 3-phosphate dehydrogenase (GAPDH) was used as a loading control.

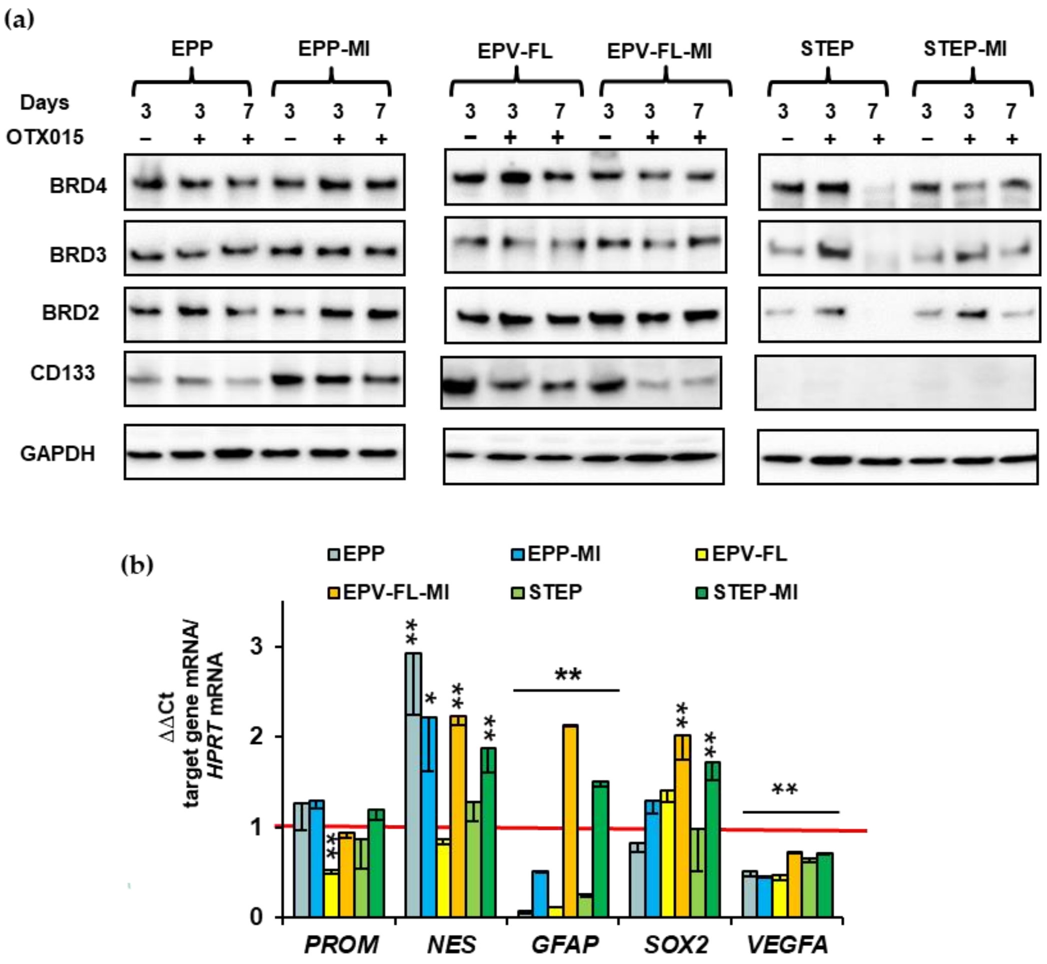


**Figure S3.** OTX015 effects on BET proteins and stemness features of EPN lines. **(a)** Western blot analysis of total lysates from EPN cell lines treated with vehicle (-) or OTX015 500 nM (+) for three and seven days. Blots were probed with the indicated antibodies. Glyceraldehide 3-phosphate dehydrogenase (GAPDH) was used as a loading control; **(b)** qPCR analysis of expression of the indicated genes. Target gene levels were normalized to the reference gene hypoxanthine-guanine phosphoribosyltransferase (*HPRT*) in each sample. Means ± SD relative to vehicle-treated controls (n = 3), which were used as calibrators (1 = no change). Student t test was used for statistical significance: *, P < 0.05; **, P < 0.01; significantly different from gene expression levels in the vehicle-treated controls. Not significant where no value is indicated.

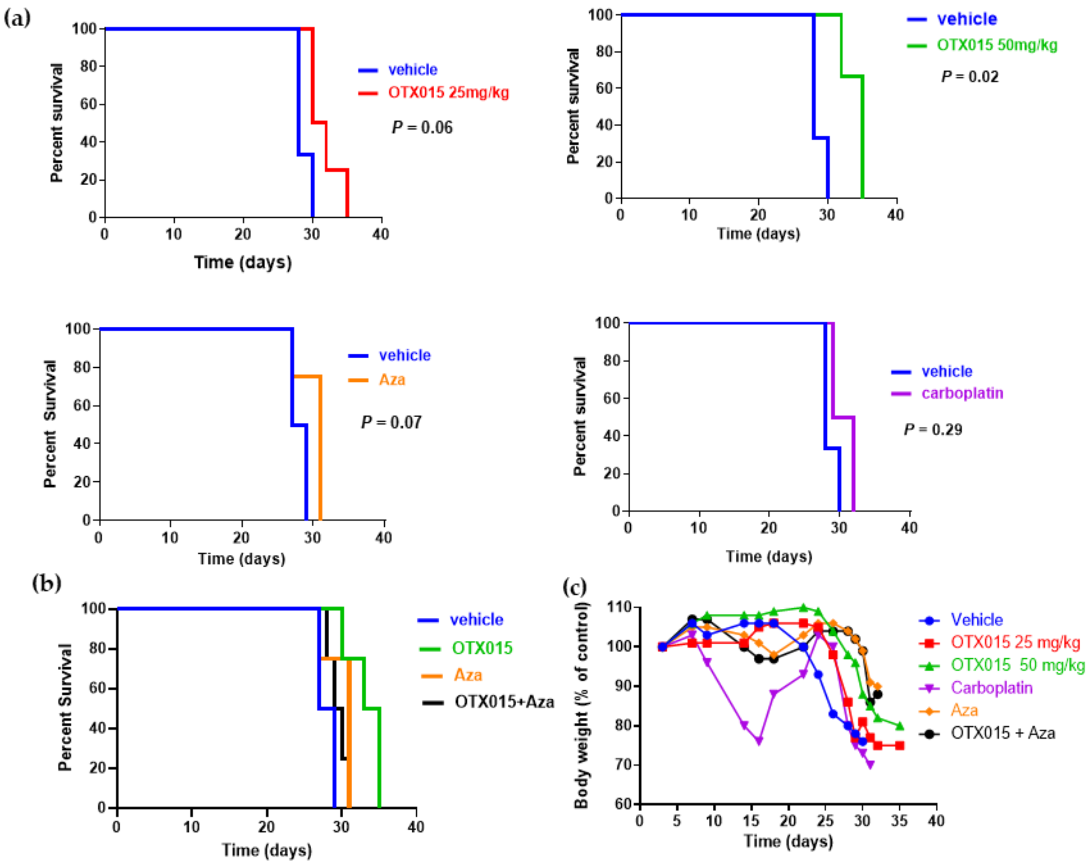


**Figure S4. (a)** Comparison of the antitumor activity of four existing therapeutic regimens in groups of mice bearing orthotopic EPP-MI xenografts. Drugs were administered as follows: OTX015 (25 or 50 mg/kg/bidaily,[ BID]), 5-azacitidine (5.0 mg/kg, intravenous [i.v.], daily for five consecutive days), carboplatin (90 mg/kg, once daily, continuous, [i.v.]), (n = 5 mice/group). Animals were sacrificed when brain tumor symptoms developed. Survival was examined using the Kaplan–Meier method. **(b)** Antitumor effects of OTX015 (50 mg/kg/BID) and Aza (5.0 mg/kg, i.v., daily for five consecutive days) were evaluated as a single agent and in combination. Survival was examined using the Kaplan–Meier method. Animals were sacrificed when brain tumor symptoms developed. Only OTX015 alone significantly prolonged the survival of the treated group with respect to the control group (log-rank P = 0.01); **(c)** Effects of the treatment regimens shown in (a) on the body weight changes of animals bearing EPP-MI xenografts.

(a)

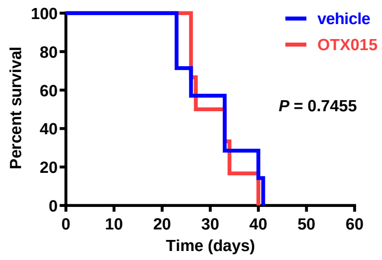


(b)

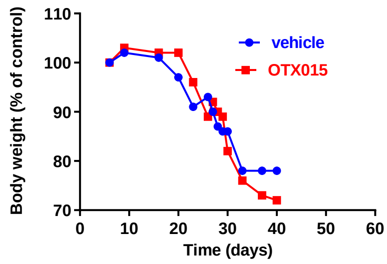


**Figure S5**. In vivo effects of OTX015 in EPV-FL-MI intracranial tumors. **(a)** Survival analysis of mice bearing EPV-FL-MI tumors. Animals were treated with vehicle or OTX015 (50 mg/kg/BID n = 5 mice/group or more). On the appearance of brain tumor symptoms, animals were sacrificed. Survival was examined using the Kaplan–Meier method; **(b)** Effects of the treatment regimen shown in panel (a) on the body weight changes of animals bearing EPV-FL-MI tumors

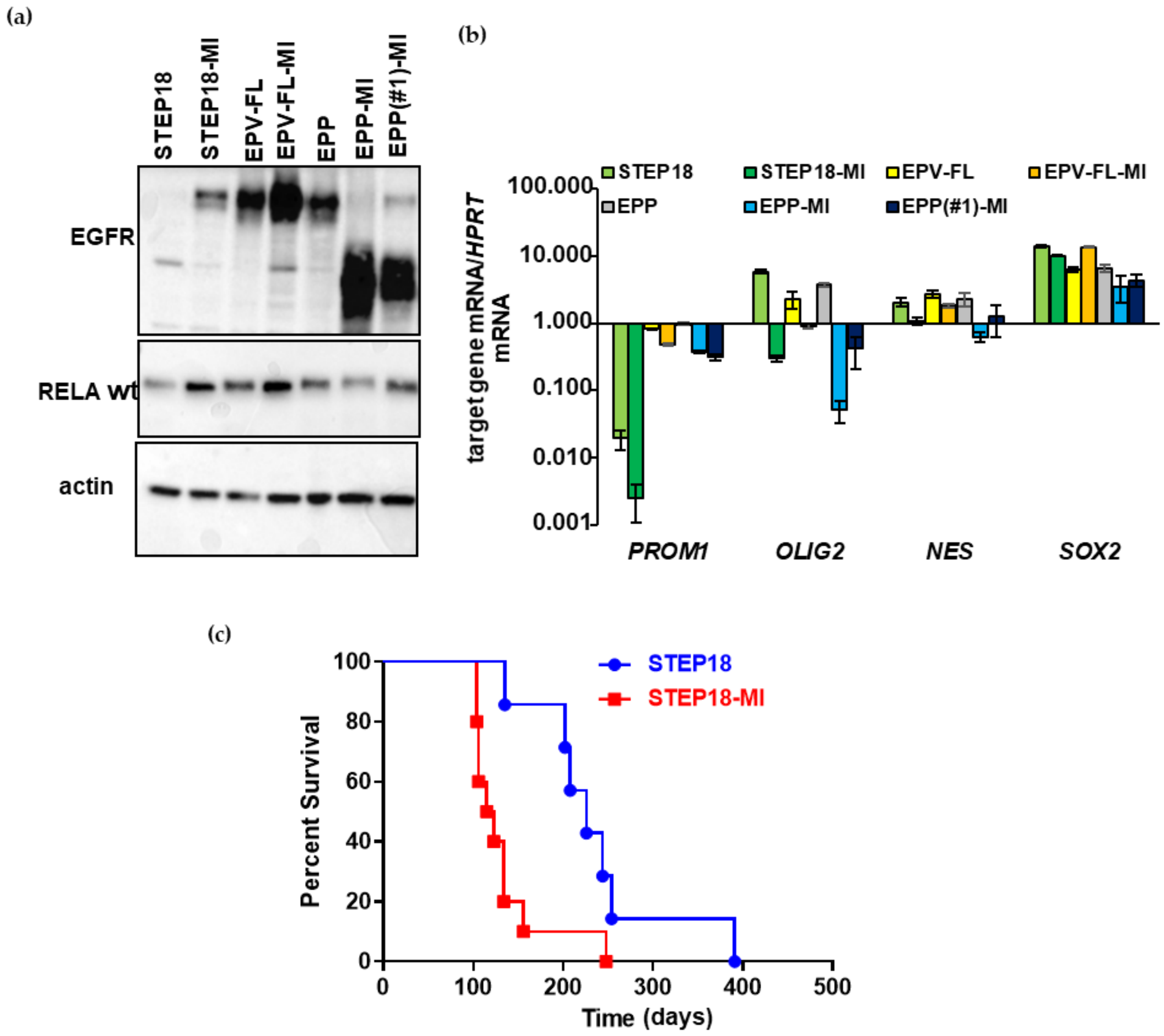


**Figure S6.** Characterization of EPN SC lines. Blots were probed with the indicated antibodies. An antibody raised against the C-terminus of epidermal growth factor receptor (EGFR) was used, that detects both wild-type EGFR and a truncated EGFR lacking the N-terminus of the receptor, which is the product of the *SEC61G-EGFR* fusion. Actin was used as a loading control. (b) qPCR analysis of expression of stemness-related genes in the panel of EPN SC-derived lines. Levels were normalized to the level of the reference gene hypoxanthine-guanine phosphorybosyltransferase (*HPRT*) in each sample. (c) Median survival of animals bearing intracranial STEP18 and STEP18-MI xenografts was 226 and 119 days, respectvely as assessed by the Kaplan-Meier method.

| Table S1. List of Antibodies used | | |
|---|---|---|
| **Antibody** | **Catalog #** | **Supplier** |
| | **Primary Antibodies** | |
| BCL2 | NCL-L-bcl-2 | CST |
| BRD2 | #5848 | CST |
| BRD3 | ab50818 | Abcam |
| BRD4 | #13440 | CST |
| caspase-3 (H-277) | sc-7148 | SCB |
| cleaved caspase-3 (Asp175) (5A1E) | #9664 | CST |
| CD133 | ab19898 | Abcam |
| cyclin D1 /HD11) | sc-246 | SCB |
| GAPDH | #5174 | CST |
| H3K27ac (D5EA) | #8173 | CST |
| H3K27me3 | #07-449 | Millipore |
| Histone 3 | #9175 | CST |
| Ki-67 | 5298512001 | Roche |
| MYC (D84C12) | #5605 | CST |
| MYCN (C-19) | sc-791 | SCB |
| NF-κB p65 (D14E121) | #8242 | CST |
| p21 Waf1/Cip1 (12D1) | #2947 | CST |
| p27 (F-8) | sc-1641 | SCB |
| p53 | sc-126 | SCB |
| Pan-actin | #4968 | CST |
| PARP | #9532 | CST |
| phospho-STAT3 (Tyr705) (D3A7) | #9145 | CST |
| STAT3 | #9139 | CST |
| survivin (D-8) | sc-17779 | SCB |
| VEGFA | EP1176 | Abcam |
| | **Secondary Antibodies** | |
| Peroxidase Anti-mouse IgG | PI-2000 | Vector |
| Peroxidase Anti-rabbit IgG | PI-1000 | Vector |
| Peroxidase Anti-goat IgG | sc-2020 | SCB |
| CST =Cell Signaling Technologies; SCB = Santa Cruz Biotechnologies | | |
